# Scissor-like Au_4_Cu_2_ Cluster with Phosphorescent Mechanochromism and Thermochromism

**DOI:** 10.3390/molecules28073247

**Published:** 2023-04-05

**Authors:** Xue-Meng Wu, Jin-Yun Wang, Ya-Zi Huang, Zhong-Ning Chen

**Affiliations:** 1 State Key Laboratory of Structural Chemistry, Fujian Institute of Research on the Structure of Matter, Chinese Academy of Sciences, Fuzhou 350002, China; 2 ShanghaiTech University, Pudong, Shanghai 201210, China; 3 University of Chinese Academy of Sciences, Beijing 100039, China; 4 Fujian Science & Technology Innovation Laboratory for Optoelectronic Information of China, Fuzhou 350108, China

**Keywords:** cluster, copper, gold, luminescence, mechanochromism, thermochromism

## Abstract

Reaction of [Au(tht)_2_](ClO_4_) (tht = tetrahydrothiophene), [Cu(CH_3_CN)_4_](ClO_4_), 3,6-di-*tert*-butyl-1,8-diethynyl-9*H*-carbazole (H_3_decz), and bis(2-diphenylphosphinophenyl)ether (POP) in the presence of triethylamine (NEt_3_) gave the cluster complex Au_4_Cu_2_(decz)_2_(POP)_2_ as yellow crystals. As revealed by X-ray crystallography, the Au_4_Cu_2_ cluster exhibits scissor-like structure sustained by two decz and two POP ligands and stabilized by Au-Cu and Au-Au interactions. The Au_4_Cu_2_ cluster shows bright yellow to orange photoluminescence upon irradiation at >300 nm, arising from ^3^[π (decz)→5d (Au)] ^3^LMCT (ligand-to-metal charge transfer) and ^3^[π→π* (decz)] ^3^IL (intraligand) triplet states as revealed by theoretical and computational studies. When it is mechanically ground, reversible phosphorescence conversion from yellow to red is observed owing to more compact molecular packing and thus stronger intermetallic interaction. Variable-temperature luminescence studies reveal that it displays distinct red-shifts of the emission whether the temperature is elevated or lowered from ambient temperature, suggestive of exceptional thermochromic phosphorescence characteristics.

## 1. Introduction

Stimuli-responsive luminescent compounds have been flourishingly developed as a class of smart solid materials with potential applications in various fields such as sensing, imaging, anti-counterfeiting, encryption, etc. [1,2,3]. Ligand-supported d^10^ metal cluster complexes are particularly attractive because of the additional d^10^-d^10^ intermetallic contacts that are extremely sensitive to external environments or stimuli and play a crucial role in modulation of photophysical properties. A number of ligand-supported d^10^ metal clusters have been found to display remarkable absorption and emission color changes upon stimulation by external conditions such as light, heat, pressure, vapor, and so on [4,5,6,7,8,9,10,11,12,13,14,15,16,17,18,19,20,21], associating mostly with the alteration of d^10^-d^10^ intermetallic contacts so that emissive energy is thus perturbed.

Among various organic ligands, alkyl or aryl ethynyls are most frequently utilized to sustain d^10^ metal cluster structures owing to the extraordinary binding capability and coordination versatility of ethynyl moieties to d^10^ metal ions through σ/π bonding, as well as diverse photophysical properties. Numerous alkyls or aryl acetylides linked d^10^ homometallic or heterometallic clusters have been acquired and characterized by X-ray crystallography, showing intriguing luminescent properties and sensitive photophysical responses to external stimulus [22,23,24,25]. Considering that bis(ethynyl) ligands are more favorable for the formation of metal cluster architectures with folding and interpenetrating topologies to afford better molecular rigidity and a stronger emissive characteristic, we are devoted to the fabrication of d^10^ metal cluster complexes using carbazole-functionalized bis(ethynyl) ligands as rigid sustaining or protective ligands [26,27]. Since diphosphines afford not only similar binding affinity to d^10^ metal ions as that of bis(ethynyl) ligands to provide exceptional metal coordination architectures but also favor improving the solubility in organic solvents, they are a class of excellent co-ligands with carbazole-functionalized bis(ethynyl) compounds for the design of d^10^ metal cluster topologies [26,27].

**Scheme 1:**
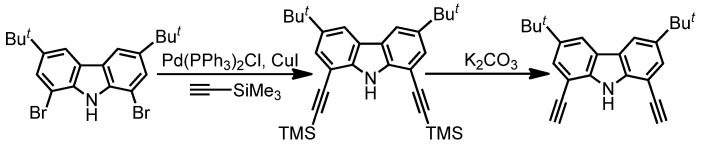
Synthetic route of ligand H_3_decz.

**Scheme 2:**
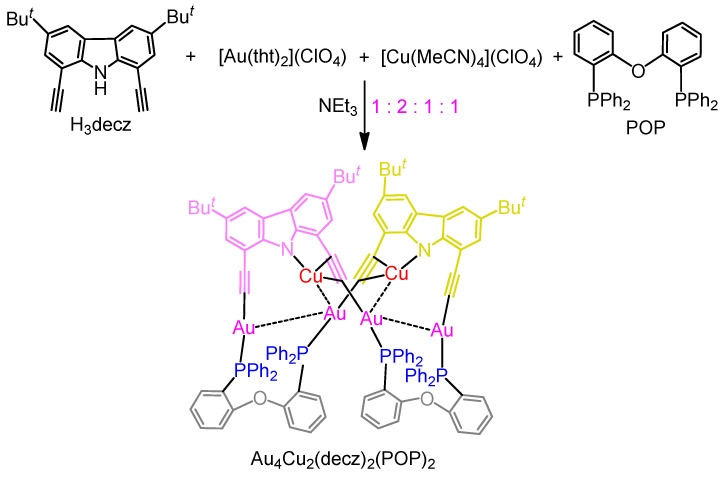
Synthetic route of Au_4_Cu_2_ cluster complex.

With 3,6-di-*tert*-butyl-1,8-diethynyl-9*H*-carbazole (H_3_decz, Scheme 1) as a rigid bis(acetylide) ligand upon deprotonation, we are able to attain a series of heterometallic cluster architectures with good molecular rigidity, impressive phosphorescent characteristics, and excellent stimuli responsiveness [6,26,27,28]. In this work, we describe a scissor-like Au_4_Cu_2_ cluster (Scheme 2) sustained by H_3_decz and bis(2-diphenylphosphinophenyl)ether (POP). It is brilliantly phosphorescent in solution and solid state at ambient temperature, originating from the ^3^LMCT (ligand-to-metal charge transfer) triplet state from decz to Au as well as the ^3^IL (intraligand) transition of decz^3−^. Interestingly, it shows remarkable phosphorescent mechanochromism and thermochromism, relevant to the variation of d^10^-d^10^ Au-Cu/Au interactions exerted by mechanical and thermal stimulus. Particularly, the observation of simultaneous red-shifts of the emission upon raising or lowering the temperature manifests its exceptional thermochromic phosphorescence characteristics.

## 2. Results and Discussion

### 2.1. Synthesis and Characterization

Ligand H_3_decz was obtained by the synthetic route shown in Scheme 1. The neutral Au_4_Cu_2_ cluster (Scheme 2) was prepared by the reactions of [Au(tht)_2_](ClO_4_), [Cu(CH_3_CN)_4_](ClO_4_), H_3_decz, and POP in a 2:1:1:1 molar ratio with the assistance of NEt_3_ for deprotonation. Re-crystallization of the product by diffusion of diethyl ether into a dimethylacetamide (DMAc) solution gave Au_4_Cu_2_(decz)_2_(POP)_2_·DMAc as yellow crystals in 50% yield. Thermogravimetric analysis (Figure S4, Supplementary Materials) revealed that solvate DMAc could be removed at a temperature range of 170–250 °C. Noticeably, the removal of solvated DMAc results in the disruption of the crystalline state to become a non-crystalline morph.

**Figure 1:**
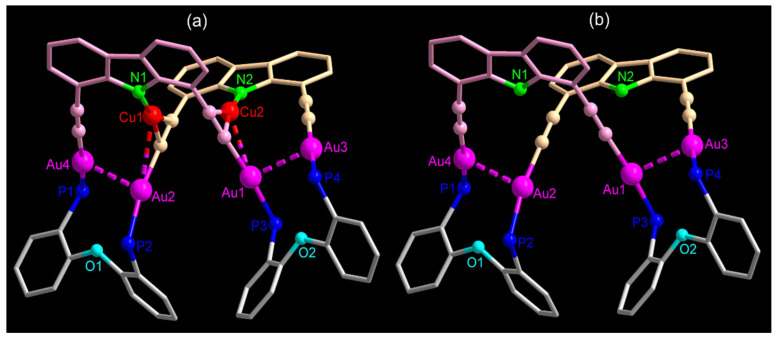
(**a**) A view of the Au_4_Cu_2_ cluster complex. The hydrogen atoms and *tert*-butyl groups, together with the phenyl rings on phosphorous atoms, were omitted for clarity. (**b**) A view showing a scissor-like structure of the Au_4_(decz)_2_(POP)_2_ framework formed by gold(I)–bis(acetylide) σ-coordination.

The structure of Au_4_Cu_2_(decz)_2_(POP)_2_·DMAc was determined by single crystal X-ray diffraction. As depicted in Figure 1, the Au_4_Cu_2_ cluster shows a scissor-like structure consisting of four Au(I) and two Cu(I) atoms linked by two tri-anionic decz^3−^ and two POP ligands through Au–acetylide σ-coordination, Cu–acetylide π-coordination, and Cu–N bonding. The Au_4_Cu_2_ cluster is further stabilized by significant d^10^-d^10^ intermetallic interactions in view of the distinctly shorter Au–Cu (2.8501(19) and 3.039(2) Å) distances than the sum of Au and Cu atoms (ca. 3.1 Å) as well as much smaller Au–Au (3.0370(7) and 3.0632(7) Å) distances than the sum of two Au atoms (3.3 Å). The Au(I) center is bound to CP donors from acetylide and POP with the C-Au-P angle of 170.7(4)–173.6(4)°. The Cu(I) center is quasi-linearly bonded to the carbazole N atom and acetylide through π-coordination. Trianionic decz^3−^ acts as a μ_4_-bridging ligand bound to two Au(I) centers through Au–acetylide σ-coordination and two Cu(I) centers through Cu–acetylide π-coordination and Cu–N bonding, respectively, strikingly different from the bonding modes reported in Ag_8_, Ag_16_, and Ag_29_ silver(I) nanoclusters as well as Ag_4_Au_6_ and Ag_8_Au_10_ heterometallic clusters sustained by H_3_decz [26,27].

The Au_4_Cu_2_ cluster was characterized by high-resolution mass spectrometry (HRMS) and ^1^H and ^31^P NMR spectroscopy. In the positive ion HRMS, the observation of the molecular ion peak [Au_4_Cu_2_(decz)_2_(POP)_2_H]^+^ together with molecular fragments [Au_4_Cu(Hdecz)_2_(POP)_2_]^+^, [Au_4_(Hdecz)_2_(POP)_2_H]^+^, and [Au_3_(decz)(POP)_2_H]^+^ suggests that the cluster structure keeps in the gas ionization state. In the ^31^P NMR spectrum, two P signals occur at 32.1 and 33.7 ppm, coinciding with the Au_4_Cu_2_ structure in a solid state.

### 2.2. Photophysical Properties and Computational Studies

**Figure 2:**
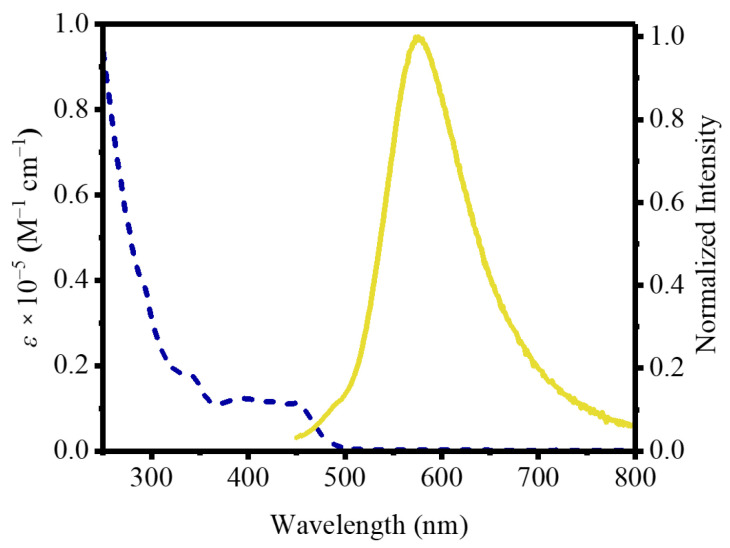
The UV-Vis absorption (blue dash) and normalized emission (yellow solid) spectra of an Au_4_Cu_2_ cluster in a CH_2_Cl_2_ solution at ambient temperature (excitation at 397 nm).

The Au_4_Cu_2_ cluster (Table 1 and Figure 2) shows intense ligand-centered absorption at <300 nm and a band at 340 nm due to metal-perturbed intraligand (IL) transitions of decz^3−^ ligands. The broad absorption in the low-energy region (380–500 nm), containing two composite bands centered at 400 and 450 nm, is mainly ascribed to the admixture of [π (decz)→5d (Au)] ^1^LMCT and [π→π* (decz)] ^1^IL states. Upon excitation at λ_ex_ > 300 nm, it exhibits bright yellow–orange luminescence peaked at 576 nm with a lifetime of 3.2 μs in degassed CH_2_Cl_2_ solution at ambient temperature, which is typical of phosphorescence in view of a large Stokes shift (Figure 2).

**Figure 3:**
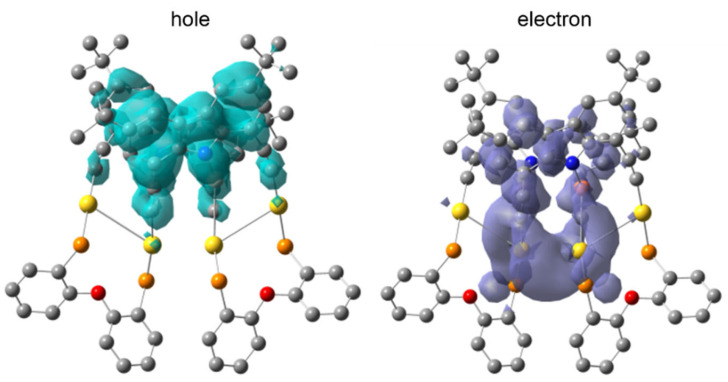
Plots of the hole and electron involved in the lowest-energy emission transition of Au_4_Cu_2_ cluster by TD-DFT method at the PBE1PBE level.

To clarify the absorption and emission origins together with the excited state character, we conducted a theoretical computational study using time-dependent density functional theory (TD-DFT) at the PBE1PBE level. In S_1_-S_5_ states (Table S3), the holes (electron-transferred moiety) focus on decz^3−^ ligands, and the electrons (electron-accepting moiety) are mostly populated at Au atoms and decz^3−^ ligands. Thus, low-energy absorption originates largely from [π (decz)→5d (Au)] ^1^LMCT and [π→π* (decz)] ^1^IL transitions. For the T_1_ state (Figure 3), since the hole and electron display a similar distribution as that in S_1_-S_5_ singlet states, the phosphorescent emission is mostly contributed by ^3^[π (decz)→5d (Au)] ^3^LMCT and ^3^[π→π* (decz^3−^)] ^3^IL triplet states, which accords with the emissive origin of Ag_4_Au_6_ and Ag_8_Au_10_ heterometallic clusters [27] sustained by H_3_decz as well as Au_4_Ag_4_ cluster complexes^15^ linked by 4,5-diethynylacridin-9-one.

**Figure 4:**
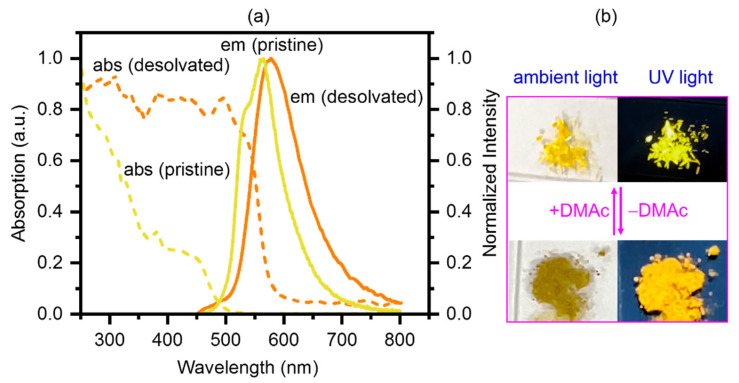
(**a**) The normalized UV-Vis absorption (dash) and emission (solid) spectra of Au_4_Cu_2_ cluster before (yellow) and after removal of solvate DMAc (orange). (**b**) The images of Au_4_Cu_2_ cluster complex under ambient light and UV light irradiation (365 nm) before and after desolvation.

Upon UV light irradiation at λ_ex_ > 300 nm, the crystalline species Au_4_Cu_2_(decz)_2_(POP)_2_·DMAc (Figure 4) shows brilliant yellow emission peaked at 565 nm with phosphorescent efficiency over 50%. When solvate DMAc was removed at 180 °C under vacuum, the species color deepened obviously, as indicated in the UV-Vis spectra (Figure 4a) and the photographs (Figure 4b) under ambient light, companying with a conversion of the crystalline to the amorphous state as revealed by X-ray diffraction. As depicted in Figure 4, the removal of solvate DMAc resulted in the emission band centered at 565 nm red-shifting distinctly to 580 nm, in which intense yellow phosphorescence converted to weaker orange emitting after desolvation. Undoubtedly, desolvation leads to more compact packing of Au_4_Cu_2_ clusters, thus lowering phosphorescent energy to some extent while enhancing thermally induced non-radiative deactivation.

### 2.3. Stimuli-Responsive Properties

**Figure 5:**
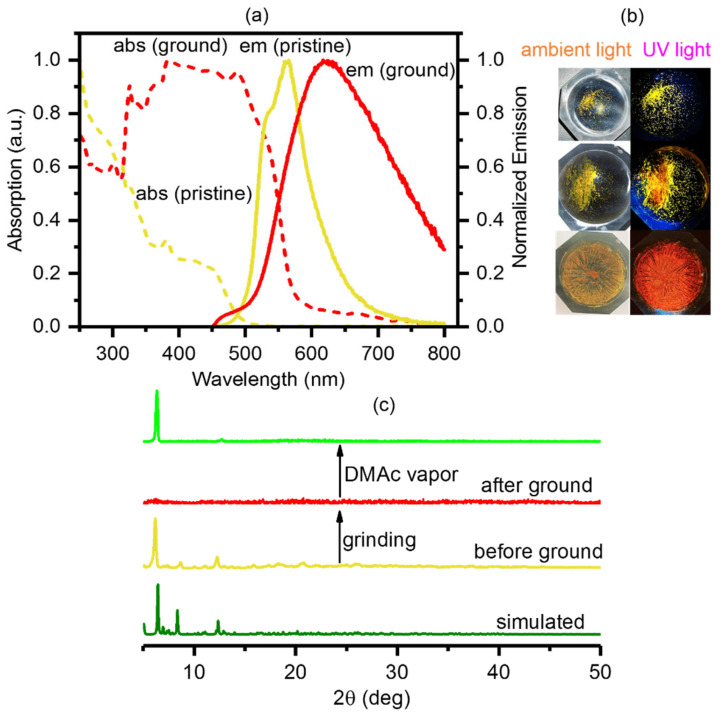
(**a**) The normalized UV-Vis absorption (dash) and emission (solid) spectra of Au_4_Cu_2_ cluster before and after mechanical grinding. (**b**) The images of Au_4_Cu_2_ cluster under ambient light and UV light irradiation (365 nm) before and after mechanical grinding. (**c**) The simulated and measured X-ray diffraction patterns of Au_4_Cu_2_ cluster upon mechanical grinding and DMAc vapor adsorption.

As shown in Figure 5, when pristine species of Au_4_Cu_2_ cluster was mechanically ground, the UV-Vis absorption showed a drastic red-shift with yellow to orange transformation under ambient light. More interestingly, upon thoroughly ground, the phosphorescent band peaked at 565 nm red-shifted dramatically to 623 nm, followed by the conversion of bright yellow to deep red emission. Noticeably, drastic red-shift of the emission gave rise to a dramatic broadening of the spectral band (Figure 5a), in which full width at half maxima (FWHM) changed from 81 nm to 196 nm upon mechanical grinding, thus manifesting more participation of metal cluster dominated triplet state. As demonstrated by TD-DFT studies, as phosphorescent emission in Au_4_Cu_2_ cluster arises mostly from admixture ^3^LMCT/^3^IL states, the dramatic broadening of the emission band after mechanical grinding is most likely ascribed to more contribution from d^10^-d^10^ intermetallic interaction whereas less component from ^3^IL state. On the other hand, when the ground species was exposed to the vapor of DMAc or CH_2_Cl_2_, the color and emission could be perfectly reverted to the original state, implying its excellent reversibility.

As shown in Figure 5c, mechanical grinding leads to the total disappearance of X-ray diffraction patterns, signifying a transformation from a crystalline to a non-crystalline state, as is observed in most metal coordination compounds with mechanochromic properties induced by intermolecular variations. When the ground species were exposed to DMAc vapor, X-ray diffraction patterns could be reverted to their original state, further demonstrating the reversibility of mechanochromism. It is known that stimuli-responsive luminescent changes triggered by intermolecular interaction are highly dependent on the doping concentration of the emitters, whereas those from an intramolecular effect are insensitive to the doping [5,6]. In order to further address this issue, a 1% Au_4_Cu_2_ cluster was doped in a PMMA matrix to explore possible machnochromism in the dilute matrix. As shown in Figure S7, the emission spectrum of 1% Au_4_Cu_2_ cluster in the PMMA matrix did not display distinct variation upon mechanical grinding, thus confirming that the phosphorescent mechanochromism of Au_4_Cu_2_ cluster results indeed from the variation of intermolecular packing instead of intramolecular effect.

**Figure 6:**
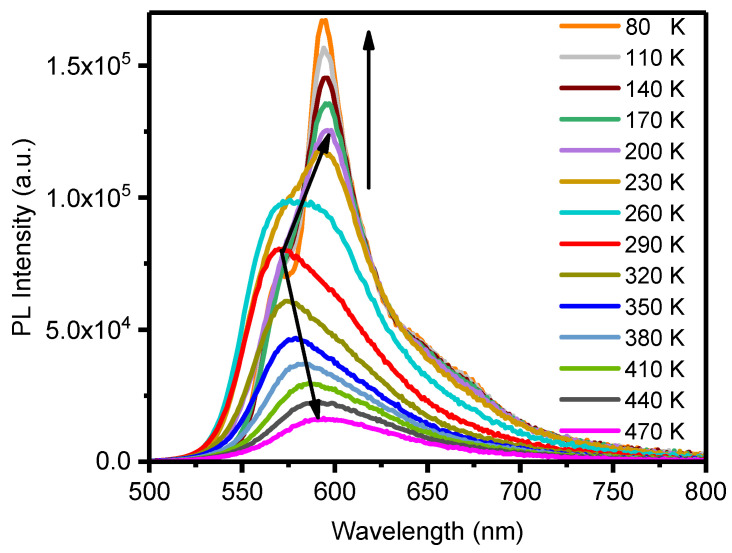
Temperature-dependent photoluminescent spectra of an Au_4_Cu_2_ cluster in solid state at 80–470 K upon excitation at 397 nm.

As depicted in Figure 6, pristine species of Au_4_Cu_2_(decz)_2_(POP)_2_·DMAc show phosphorescent emission centered at ca. 570 nm at an ambient temperature of 290 K. When the temperature was step by step raised to 470 K, the emission band followed 570 nm (290 K)→575 nm (320 K)→578 nm (350 K)→581 nm (380 K)→584 nm (410 K)→587 nm (440 K)→590 nm (470 K) to show a gradual red-shift with a progressive reduction of the intensity. The stepwise red-shift with temperature raising is ascribed to the gradual desolvation process as discussed above, whereas a progressive decrease in the emission intensity is due to the increasingly increased thermally non-radiative deactivation process. On the other hand, when the temperature is lowered to liquid nitrogen, the emission follows 570 nm (290 K)→580 nm (260 K)→593 nm (230 K)→595 nm (200 K) to show a gradual red-shift, which is assignable to the stepwise shortening of intermetallic distances and the successive increase in d^10^-d^10^ interaction so that phosphorescent energy is progressively reduced. The consecutive emission enhancement is due to the step-by-step suppression of thermal vibration and, thus, the reduced non-radiative deactivation with the temperature progressively lowered to the liquid nitrogen range.

## 3. Experimental

All operations were carried out under a dry argon atmosphere using Schlenk techniques and vacuum-line systems. The solvents were dried, distilled, and degassed prior to use. Spectroscopic-grade reagents for spectroscopic measurements were purchased from commercial sources. Bis(2-diphenylphosphinophenyl)ether (POP), 1,8-dibromo-3,6-di-*tert*-butylcarbazole, and other reagents were purchased from commercial sources without further purification. Perchlorate salts are potentially explosive and should be used carefully.

### 3.1. Synthesis of Ligand H_3_decz

H_3_decz was prepared by the modified procedures described in the literature [29]. Additionally, 1,8-dibromo-3,6-di-*tert*-butylcarbazole (4.7 mmol, 2.0 g), Pd(PPh_3_)_2_Cl_2_ (0.3 mmol, 0.2 mg), CuI (0.3 mmol, 57.4 mg), ethynyltrimethylsilane (13 mmol, 5.0 g), and NEt_3_ (30 mL) were put into a high-pressure flask. After heating at 90 °C for 2 d, the black precipitate was removed through filtration. The filtrate was evaporated in a vacuum to give a brown solid after the removal of the solvent. The product was then purified through silica gel column chromatography using petroleum ether as an eluent, giving 3,6-di-*tert*-butyl-1,8-bis((trimethylsilyl)ethynyl)-9*H*-carbazole as a yellow solid. Yield: 75%.

Then, 3,6-di-*tert*-butyl-1,8-bis((trimethylsilyl)ethynyl)-9*H*-carbazole (10 mmol, 4.3 g) and K_2_CO_3_ (10 mmol, 1.4 g) were added to MeOH-CH_2_Cl_2_ (100 mL, *v*/*v* = 1:1). Upon stirring at ambient temperature for 10 h, the solvents were removed under vacuum. The crude product was chromatographed on a silica gel column using petroleum ether as an eluent to give H_3_decz as a pale yellow solid. Yield: 80%. ^1^H NMR (CDCl_3_, ppm): 8.50 (s, 1H), 8.13 (d, 2H, *J* = 7.8 Hz), 7.68 (d, 2H, *J* = 8.1 Hz), 3.49 (s, 2H), and 1.48 (s, 18H). ^13^C NMR (CDCl_3_, ppm): 142.71, 139.16, 127.49, 123.22, 117.95, 103.87, 81.37, 80.45, 34.69, and 31.87.

### 3.2. Synthesis of Au_4_Cu_2_(decz)_2_(POP)_2_

Freshly prepared [Au(tht)_2_](ClO_4_) (0.08 mmol, 37.8 mg) was obtained by mixing an equivalent molar of Ag(tht)(ClO_4_) (0.08 mmol, 23.6 mg) and Au(tht)Cl (0.08 mmol, 18.5 mg) in 10 mL CH_2_Cl_2_ with stirring for 10 min. The turbid solution was treated by centrifugation to remove the AgCl precipitate, giving a clear solution that was used directly in the next step.

To the filtrate was added [Cu(MeCN)_4_](ClO_4_) (0.04 mmol, 13.1 mg), POP (0.04 mmol, 21.6 mg), and H_3_decz (0.04 mmol, 13.1 mg). Upon stirring for 5 min, NEt_3_ (0.5 mL) was added to the above solution, and the mixture immediately turned from yellow to dark red. After the mixed solution was stirred overnight in the dark, the solvent was removed under a vacuum to give a yellow solid. The solid was re-dissolved in 4 mL of dimethylacetamide (DMAc). When the clear solution was exposed to diethyl ether for slow vapor diffusion, yellow crystals were grown in one week. Yield: 50%. HRMS *m*/*z* (%): 2641.3815 (6) [Au_4_Cu_2_(decz)_2_(POP)_2_H]^+^ (Calcd 2641.4063), 2579.5228 (37) [Au_4_Cu(Hdecz)_2_(POP)_2_]^+^ (Calcd. 2579.4483), 2516.5594 (34) [Au_4_(Hdecz)_2_(POP)_2_H]^+^ (Calcd. 2516.5677), 1993.4868 (100) [Au_3_(decz)(POP)_2_H]^+^ (Calcd. 1993.4131). Anal. Calcd for C_120_H_100_Au_4_Cu_2_N_2_O_2_P_4_: C, 54.57; H, 3.82; N, 1.06. Found: C, 54.39; H, 3.80; N, 0.97. ^1^H NMR (400 MHz, DMSO-*d*_6_, δ): 8.26 (s, 1H), 8.24 (s, 1H), 8.23 (s, 1H), 8.21 (s, 1H), 8.12 (d, *J* = 4.2 Hz, 2H), 8.09 (s, 2H), 8.06 (s, 1H), 8.05 (s, 1H), 7.95 (s, 1H), 7.95 (s, 1H), 7.76 (d, *J* = 2.0 Hz, 4H), 7.74 (s, 4H), 7.70 (d, *J* = 2.3 Hz, 4H), 7.47–7.36 (m, 12H), 7.35 (s, 2H), 7.28 (dd, *J* = 8.2, 4.9 Hz, 3H), 7.14 (d, *J* = 8.3 Hz, 8H), 6.86 (t, *J* = 7.5 Hz, 3H), 6.71 (d, *J* = 7.9 Hz, 2H), 6.69–6.66 (m, 2H), 6.50 (d, *J* = 7.9 Hz, 3H), 6.06–6.00 (m, 3H), 5.32 (t, *J* = 4.9 Hz, 2H), 1.37 (s, 18H), 1.06 (s, 18H). ^31^P NMR (162 MHz, DMSO-*d*_6_): 32.1 (2P), 33.7 (2P). IR (KBr, cm^−1^): 2105w (C≡C).

### 3.3. Physical Measurements

The UV-Vis absorption spectra were recorded on a Perkin-Elmer Lambda 365 UV-Vis spectrophotometer (Perkin-Elmer, Waltham, MA, USA). The infrared spectra (IR) were conducted on a Bruker VERTEX 70 FT-IR spectrophotometer (Bruker, Mannheim, Germany) with KBr pellets. The high-resolution mass spectrometry (HRMS) was performed on a Waters Xevo G2-Q-Tof mass spectrometer (Waters, Milford, MA, USA) using dichloromethane and methanol mixtures as mobile phases. The ^1^H NMR spectra were recorded on a Bruker Avance III (400 MHz) spectrometer (Bruker, Zurich, Switzerland) using SiMe_4_ as the internal reference, and ^31^P NMR spectra were measured using H_3_PO_4_ as the external reference. The excitation and emission spectra, together with emissive lifetimes in degassed solutions, solid states, and films, were determined on an Edinburgh FLS1000 fluorescence spectrometer (Edinburgh Instruments, Livingston, UK). Absolute quantum yields were determined by the integrating sphere (142 mm in diameter) using the Edinburgh FLS1000 Spectrofuorophotometer (Edinburgh Instruments, Livingston, UK). The PMMA doping was conducted by mixing 99% PMMA and 1% Au_4_Cu_2_ complex in CH_2_Cl_2_. The diluted species obtained after the removal of the solvent were thus used for photophysical studies.

### 3.4. Crystal Structural Determination

Crystals suitable for X-ray crystallographic measurement were grown by vapor diffusing of diethyl ether to a DMAc solution. The single crystal X-ray diffraction was carried out on a micro-focus metaljet diffractometer using Ga Kα radiation (λ = 1.3405 Å). Data reduction was conducted with the CrysAlisPro package, and at last an analytical absorption correction was performed. The structure was determined using the Bruker SHELXTL Software Package, a computer program for the automatic solution of crystal structures. The structure was refined by the full-matrix least-square method with ShelXle Version 4.8.6, a Qt graphical user interface for the SHELXL [30,31]. All non-hydrogen atoms were refined anisotropically, whereas the hydrogen atoms were generated geometrically and refined isotropically. CCDC 2245976 contains the supplementary crystallographic data for this paper. These data can be obtained free of charge from the Cambridge Crystallographic Data Centre via www.ccdc.cam.ac.uk/data_request/cif (accessed on 3 March 2023).

### 3.5. Computational Method

The computational studies were carried out using the Gaussian 16 program package [32]. The geometrical structures in the ground state and the lowest-energy triplet state were optimized respectively by the restricted and unrestricted density functional theory (DFT) method by the use of the gradient-corrected correlation functional PBE1PBE [33,34] and the D3 version of Grimme’s dispersion with the original D3 damping function [35] (here abbreviated as PBE1PBE-GD3). The initial structure of the Au_4_Cu_2_ cluster was obtained from crystal structural data. To decrease the computation time, the structure was simplified by replacing the phenyl rings on phosphorous atoms with H atoms. To analyze the absorption and emission transition properties, the calculations were conducted to give 60 singlet and 6 triplet excited states based on the optimized structures in the ground state and lowest-energy triplet state, respectively, to determine the vertical excitation energies by time-dependent density functional theory (TD-DFT) [36,37,38] at the PBE1PBE-GD3 level. The polarizable continuum model method (PCM) [39] with CH_2_Cl_2_ as solvent was utilized for the calculation of excited states. In these calculations, we used the Stuttgart-Dresden (SDD) basis set with the f-type polarization functions (f-exponent is 1.050 for Au and 3.525 for Cu atoms) and the effective core potentials (ECPs) to describe the Au and Cu atoms [40,41]. We used the all-electron basis set of 6-31G** to describe other non-metal atoms, including P, O, N, C, and H. We performed visualization of the hole and electron plots using GaussView, and we analyzed the contributions of fragments to the hole and electron in the electronic excitation process using the Multiwfn 3.8 program [42].

## 4. Conclusions

We obtain an Au_4_Cu_2_ cluster based on carbazole-bis(acetylide) as a supporting platform, which shows a scissor-like structure as demonstrated by X-ray crystallography. The Au_4_Cu_2_ cluster shows a yellow to red phosphorescence transformation upon mechanical grinding, and red emission could be reversibly reverted to yellow emitting through DMAc vapor adsorption. The observation of distinct red-shifts of the emission upon not only raising but also lowering the temperature at ambient temperature reveals an exceptional phosphorescent thermochromism due to different mechanisms. This work provides a vivid example of stimuli-responsive modulation of phosphorescent properties in smart solid materials taking advantage of d^10^-d^10^ intermetallic interactions.

## Figures and Tables

**Table 1:** The UV-Vis absorption and luminescent data of Au_4_Cu_2_ cluster at ambient temperature.

*λ*_abs_/nm (*ε*/M^−1^ cm^−1^)	*λ*_em_ (nm)/*τ*_em_ (μs)/*Φ*_em_ (%)		
CH_2_Cl_2_	CH_2_Cl_2_ ^a^	Solid ^b^	PMMA Film ^c^
290 (39680), 342 (17540), 400 (12200), 450 (11200)	576/2.6/3.2	565/14.5/50.2 580/6.0/1.2 (desolvated)	530/19.3/7.9
	580/1.9/3.6	623/5.3/12.6 (ground)	529/16.7/5.6 (ground)

^a^ Measured in degassed CH_2_Cl_2_ solutions upon excitation at 397 nm. ^b^ Excitation at 397 nm. ^c^ Consisting of 99% PMMA and 1% Au_4_Cu_2_ complex upon excitation at 397 nm.

## Data Availability

All data needed to support the conclusions of this manuscript are included in the main text or the Supplementary Materials.

## Supplementary Materials

The following supporting information can be downloaded at: https://www.mdpi.com/article/10.3390/molecules28073247/s1, Table S1: Crystallographic Data for Au_4_Cu_2_ Cluster Complex; Table S2: Selective Interatomic Distances (Å) and Bonding Angles (°) of Au_4_Cu_2_ Cluster Complex; Table S3: The Absorption Transitions for Au_4_Cu_2_ Cluster Complex in CH_2_Cl_2_ solution, Calculated by TD-DFT Method at the PBE1PBE Level (isovalue = 0.0004); Table S4: The Emission Transitions for Au_4_Cu_2_ Cluster Complex in CH_2_Cl_2_ Solution, Calculated by TD-DFT Method at the PBE1PBE Level (isovalue = 0.0004); Figure S1: The high-resolution mass spectrometry of Au_4_Cu_2_ cluster complex; Figure S2: The ^1^H NMR spectrum of Au_4_Cu_2_ cluster complex in DMSO-*d_6_* solution at ambient temperature; Figure S3: The ^31^P NMR spectrum of Au_4_Cu_2_ cluster complex in DMSO-*d* solution at ambient temperature; Figure S5: The measured (solid) and calculated (column bar) UV-Vis absorption spectra for Au_4_Cu_2_ cluster complex by TD-DFT method at the PBE1PBE level; Figure S6: Plots of the simulated and measured X-ray diffraction patterns of Au_4_Cu_2_ cluster complex before and after desolvation; Figure S7: The normalized emission spectra of 1% Au_4_Cu_2_ cluster complex in PMMA matrix before and after mechanical grinding (excitation at 397 nm); Figure S8: The normalized UV-Vis absorption (dash) and photoluminescent (solid) spectra of Au_4_Cu_2_ cluster in as-prepared pristine state, desolvation and mechanical grinding.

## Sample Availability

Samples of the compounds are available from the authors.
