# Supplementary material for: Scissor-like Au4Cu2 Cluster with Phosphorescent Mechanochromism and Thermochromism
Source: Molecules. 2023 Apr 5;28(7):3247. doi: 10.3390/molecules28073247 (PMC10096801; doi:10.3390/molecules28073247)
Supplement: Supplementary file 1 [file molecules-28-03247-s001.zip › molecules-2293768-supplementary.pdf]

Supplementary Materials

Scissor-like Au_4Cu_2 Cluster with Phosphorescent Mechanochromism and Thermochromism

Xue-Meng Wu [1,2,3], Jin-Yun Wang [1], Ya-Zi Huang [1] and Zhong-Ning Chen[1,2,3,4,*]

[1] *State Key Laboratory of Structural Chemistry, Fujian Institute of Research on the Structure of Matter, Chinese Academy of Sciences, Fuzhou 350002, China*

[2] *ShanghaiTech University, 393 Middle Huaxia Road, Pudong, Shanghai, 201210, China*

[3] *University of Chinese Academy of Sciences, Beijing 100039, China*

[4] *Fujian Science & Technology Innovation Laboratory for Optoelectronic Information of China, Fuzhou, Fujian 350108, China*

** Correspondence: czn@fjirsm.ac.cn*

Table S1. Crystallographic Data for Au_4Cu_2 Cluster Complex.

empirical formula	$C_{120}H_{100}Au_4Cu_2N_2O_2P_4 \cdot C_4H_9NO$
formula weight	2727.96
crystal system	monoclinic
space group	$P2_1/c$
a (Å)	17.4719(9)
b (Å)	22.7390(16)
c (Å)	27.536(2)
β (deg)	94.451(6)
V (Å^3)	10906.8(13)
Z	4
F (000)	5328
completeness	0.986
ρ_{calcd} (g/cm^3)	1.661
μ (mm^{-1})	9.482
radiation (λ, Å)	1.3405
temperature (K)	100(2)
GOF	1.004
R1 (F_o)[a]	0.0667
wR2 (F_o^2)[b]	0.1508

[a] $R1 = \Sigma|F_o - F_c|/\Sigma F_o$ [b] $wR2 = \Sigma[w(F_o^2 - F_c^2)^2]/\Sigma[w(F_o)^2)]^{1/2}$

Table S2. Selective Interatomic Distances (Å) and Bonding Angles (°) of Au_4Cu_2 Cluster Complex.

interatomic distance			
Au1–Au3	3.0371(7)	Au1–C25	2.002(16)
Au1–Cu2	2.8501 (19)	Au1–P3	2.268(3)
Au2–Au4	3.0632 (7)	Au2–C13	2.032(16)
Au2–Cu1	3.039 (2)	Au2–P2	2.262(3)
Au3–C1	2.008 (13)	Au3–P4	2.279(3)
Au4–C44	1.998 (14)	Au4–P1	2.271(3)
bonding angle			
C25–Au1–Au3	89.2 (4)	C25–Au1–Cu2	42.9 (4)
C25–Au1–P3	173.6 (4)	Cu2–Au1–Au3	94.42(4)
P3–Au1–Au3	97.15 (8)	P3–Au1–Cu2	135.35 (9)
C13–Au2–Au4	78.8(3)	C13–Au2–Cu1	37.9 (4)
C13–Au2–P2	170.7 (4)	Cu1–Au2–Au4	84.65(4)
P2–Au2–Au4	109.17 (8)	P2–Au2–Cu1	145.06(9)
C1–Au3–Au1	77.6 (3)	C1–Au3–P4	173.1(4)
P4–Au3–Au1	106.61(8)	C44–Au4–Au2	81.9 (4)
C44–Au4–P1	171.4 (4)	P1–Au4–Au2	106.43 (8)
C2–C1–Au3	177.2 (11)		

Table S3. The Absorption Transitions for Au_4Cu_2 Cluster Complex in CH_2Cl_2 solution, Calculated by TD-DFT Method at the PBE1PBE Level (isovalue = 0.0004).

state	*E*, nm (eV)	O.S.	transition (Contrib.)	hole	electron	assignment
				Au / Cu / POP / decz (%)		
S_1	480 (2.58)	0.0099	HOMO→LUMO (97%)	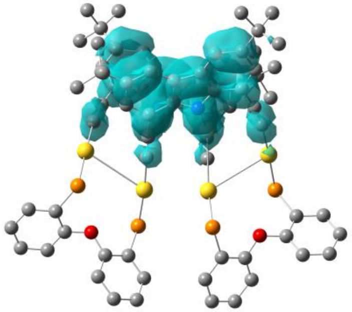 1.68/10.36/0.25/87.71	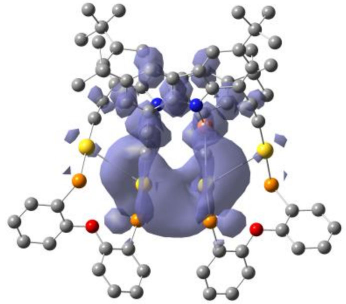 37.56/6.33/17.74/38.37	^{1}LMCT/^{1}IL
S_2	456 (2.72)	0.0336	H-1→LUMO (94%)	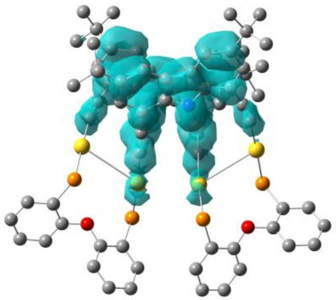 6.29/11.87/0.86/80.97	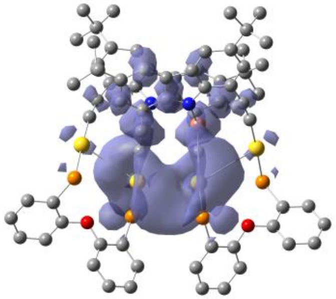 39.24/5.72/18.71/36.33	^{1}LMCT/^{1}IL
S_3	433 (2.86)	0.1684	HOMO→L+1 (92%)	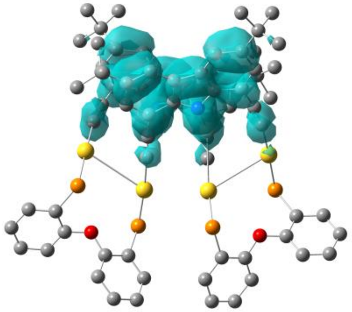 1.73/9.86/0.25/88.16	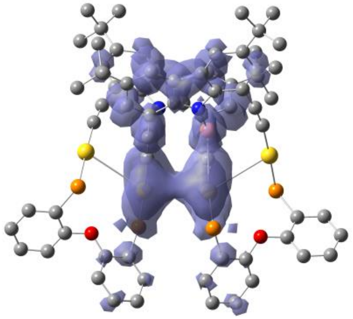 25.34/5.96/15.53/53.17	^{1}IL/^{1}LMCT

S_4	422 (2.94)	0.0936	HOMO→L+2 (80%)<br>HOMO→L+4 (11%)	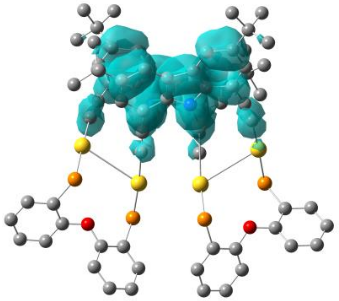<br>1.8/9.61/0.26/88.33	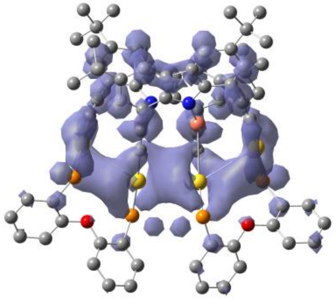<br>31.66/2.58/23.42/42.34	^{1}IL/^{1}LMCT/^{1}LLCT
S_5	412 (3.01)	0.2305	H-1→L+1 (91%)	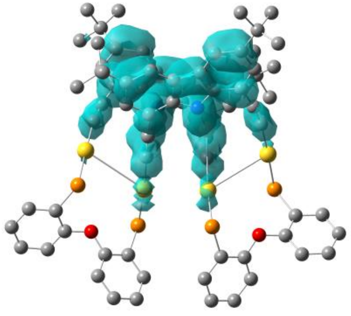<br>5.37/9.8/0.78/84.05	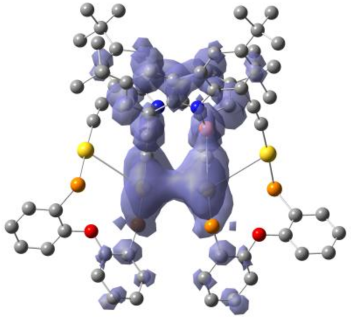<br>25.61/6.01/18.73/49.65	^{1}IL/^{1}LMCT/^{1}LLCT
S_{30}	329 (3.77)	0.0535	H-2→L+1 (56%)<br>H-4→L+1 (16%)	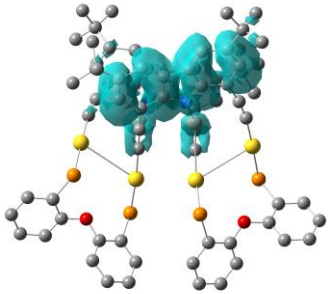<br>0.41/2.72/0.05/96.83	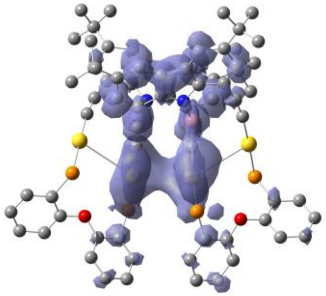<br>26.6/4.68/17.59/51.13	^{1}IL/^{1}LMCT/^{1}LLCT

S_{31}	325 (3.82)	0.0839	H-5→L+1 (36%)<br>H-3→L+1 (27%)<br>H-4→L+1 (10%)	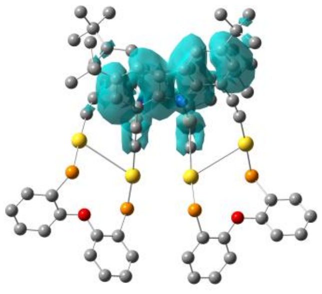<br>0.8/5.5/0.08/93.63	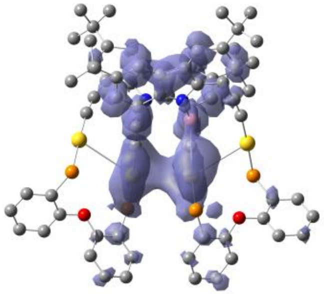<br>26.73/4.74/18.21/50.32	^{1}IL/^{1}LMCT/^{1}LLCT

Table S4. The Emission Transitions for Au_4Cu_2 Cluster Complex in CH_2Cl_2 Solution, Calculated by TD-DFT Method at the PBE1PBE Level (isovalue = 0.0004).

state	E, nm (eV)	O.S.	transition (Contrib.)	hole	electron	assignment
				Au / Cu / POP / decz (%)		
T_1	584 (2.12)	0.0000	HOMO→LUMO (77%)	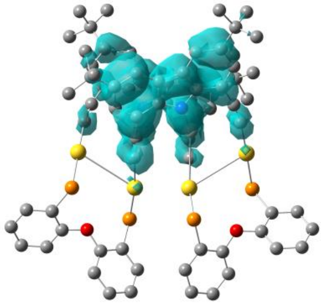<br>2.4/11.73/0.34/85.53	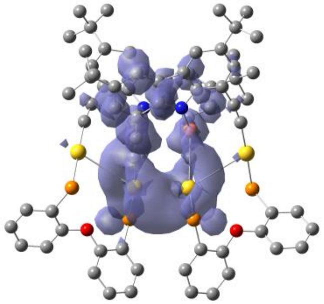<br>31.17/6.13/13.97/48.74	^{3}LMCT/^{3}IL

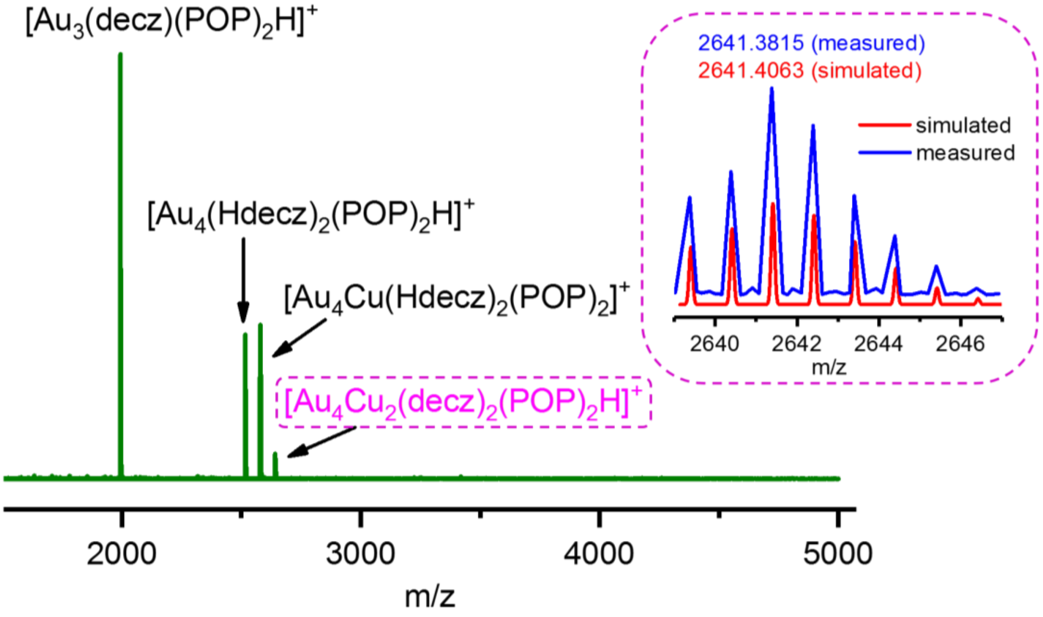

Figure S1. The high-resolution mass spectrometry of Au_4Cu_2 cluster complex. Inset: The measured and simulated isotopic patterns.

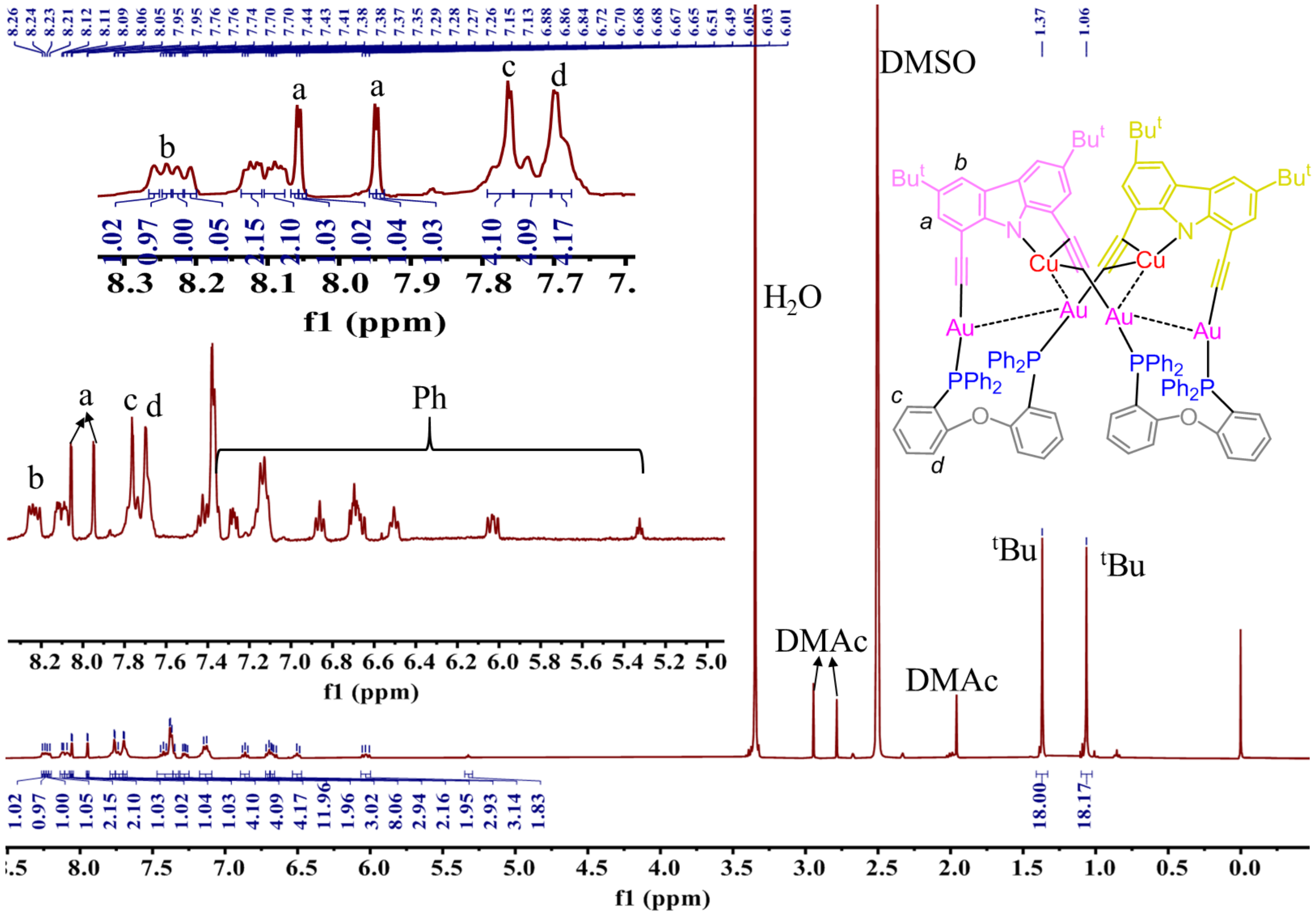

Figure S2. The ^1H NMR spectrum of Au_4Cu_2 cluster complex in DMSO-d_6 solution at ambient temperature.

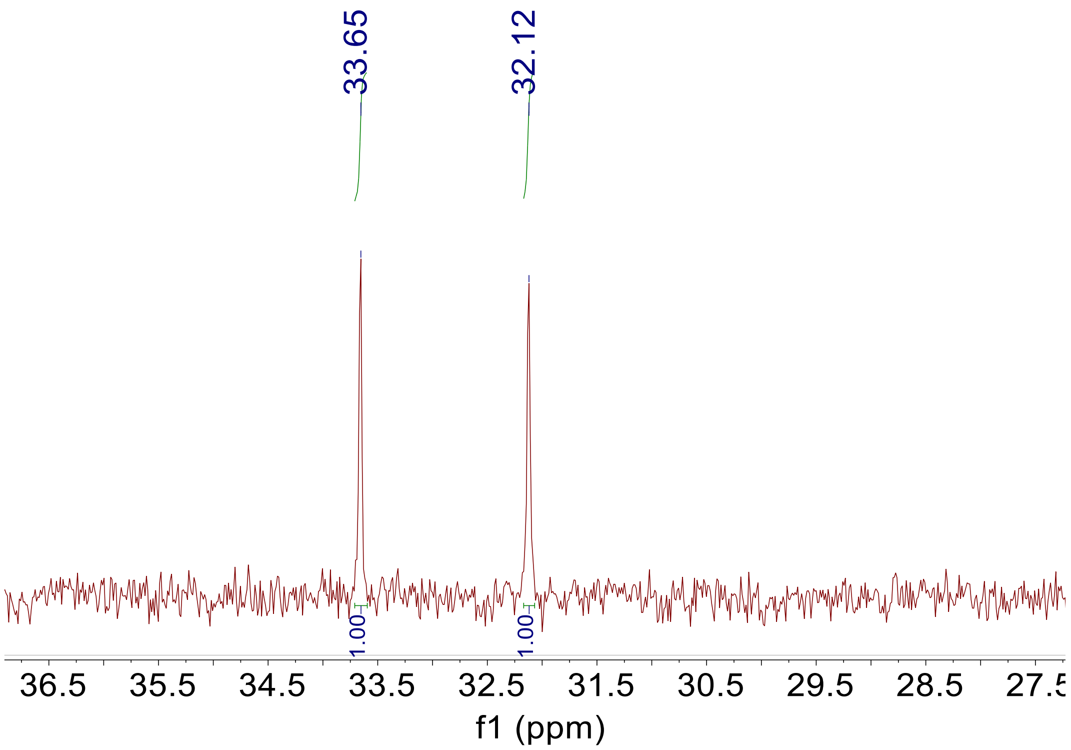

Figure S3. The ^{31}P NMR spectrum of Au_4Cu_2 cluster complex in DMSO-d_6 solution at ambient temperature.

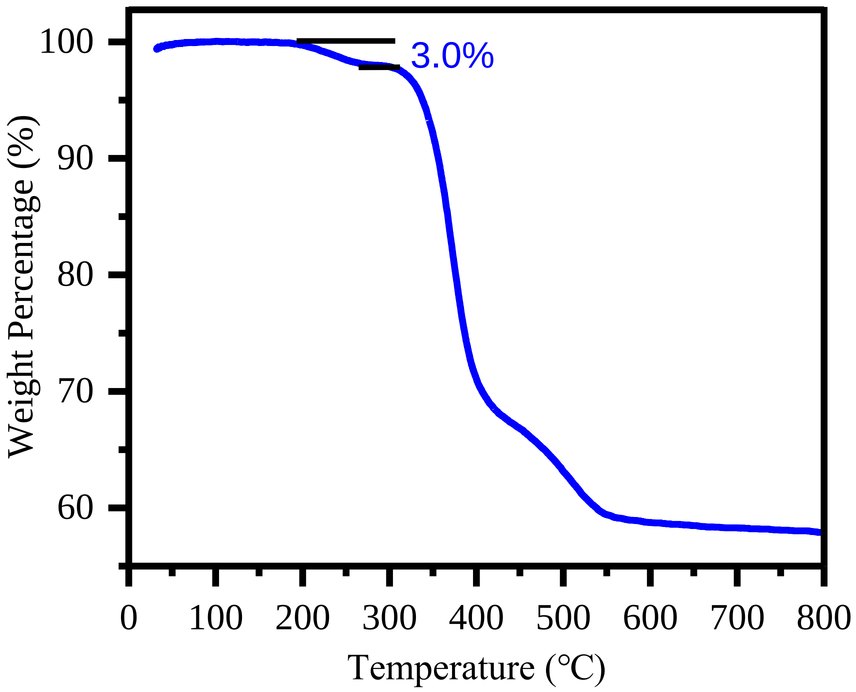

Figure S4. The plot of thermogravimetric analysis of Au_4Cu_2 cluster complex.

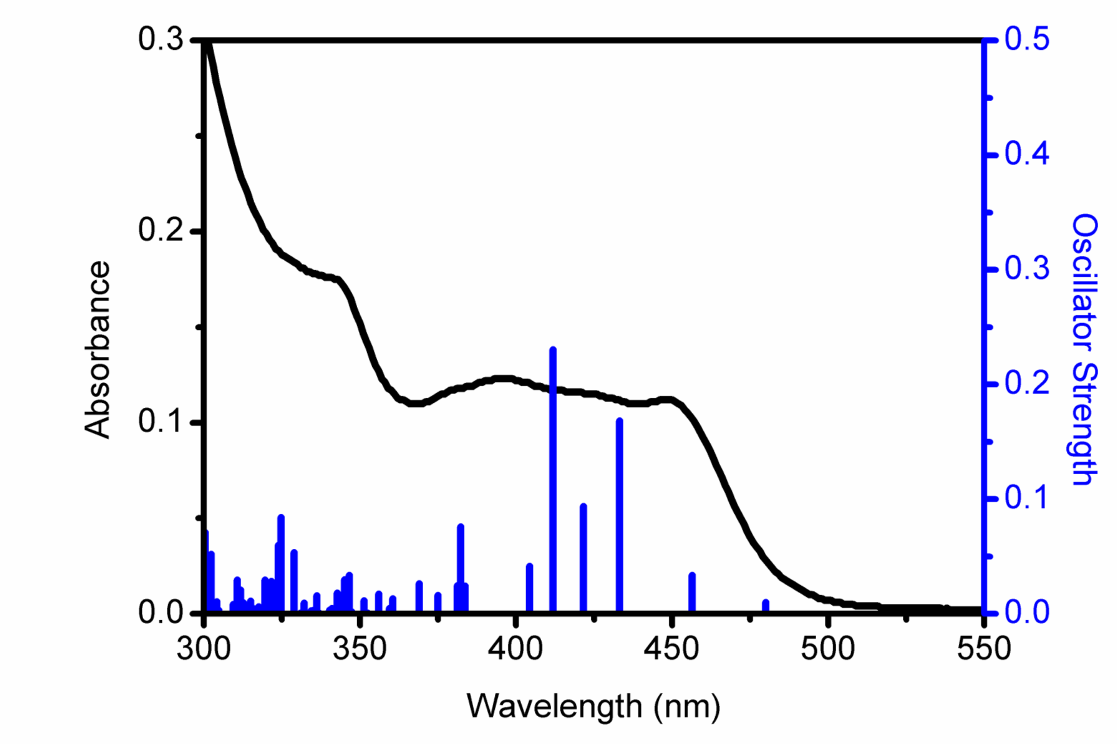

Figure S5. The measured (solid) and calculated (column bar) UV-Vis absorption spectra for Au_4Cu_2 cluster complex by TD-DFT method at the PBE1PBE level.

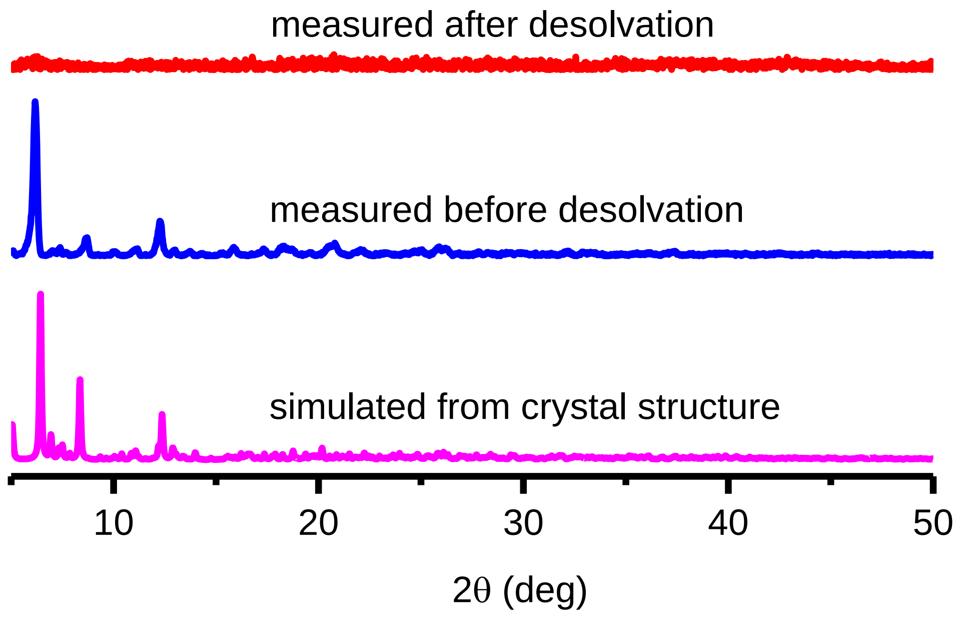

Figure S6. Plots of the simulated and measured X-ray diffraction patterns of Au_4Cu_2 cluster complex before and after desolvation.

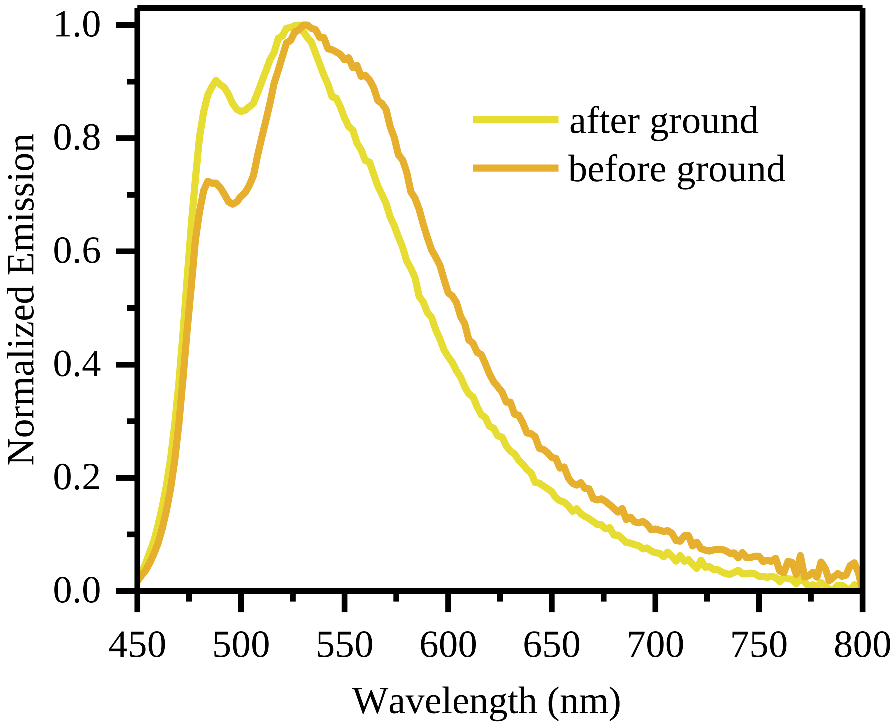

Figure S7. The normalized emission spectra of 1% Au_4Cu_2 cluster complex in PMMA matrix before and after mechanical grinding (excitation at 397 nm).

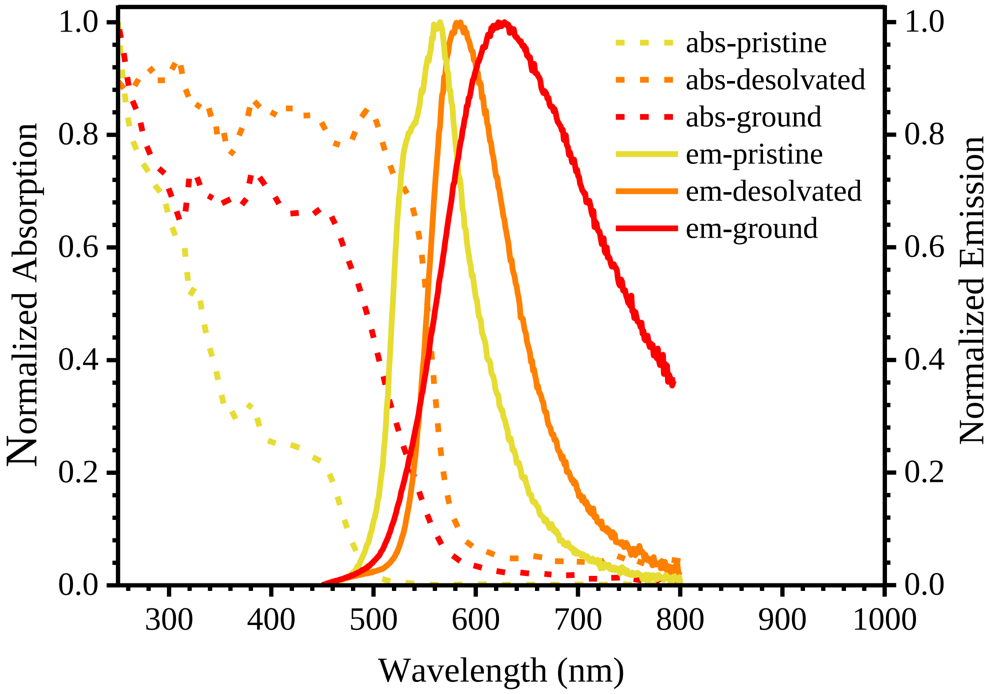

Figure S8. The normalized UV-Vis absorption (dash) and photoluminescent (solid) spectra of Au_4Cu_2 cluster in as-prepared pristine state, desolvation and mechanical grinding.
